# The State of Immunotherapy in Hepatobiliary Cancers

**DOI:** 10.3390/cells10082096

**Published:** 2021-08-15

**Authors:** Farhan Z. Ilyas, Joal D. Beane, Timothy M. Pawlik

**Affiliations:** 1 College of Medicine, The Ohio State University Wexner Medical Center, Columbus, OH 43210, USA; Farhan.Ilyas@osumc.edu; 2 Department of Surgery, The Ohio State University Wexner Medical Center, Columbus, OH 43210, USA; Joal.Beane@osumc.edu; 3 The James Comprehensive Cancer Center, Columbus, OH 43210, USA

**Keywords:** hepatocellular carcinoma, cholangiocarcinoma, gallbladder carcinoma, immunotherapy, immune checkpoint inhibitor, adoptive cell transfer, tumor vaccine

## Abstract

Hepatobiliary cancers, including hepatocellular carcinoma (HCC), cholangiocarcinoma (CCA), and gallbladder carcinoma (GBC), are lethal cancers with limited therapeutic options. Curative-intent treatment typically involves surgery, yet recurrence is common and many patients present with advanced disease not amenable to an operation. Immunotherapy represents a promising approach to improve outcomes, but the immunosuppressive tumor microenvironment of the liver characteristic of hepatobiliary cancers has hampered the development and implementation of this therapeutic approach. Current immunotherapies under investigation include immune checkpoint inhibitors (ICI), the adoptive transfer of immune cells, bispecific antibodies, vaccines, and oncolytic viruses. Programmed cell death protein 1 (PD-1) and cytotoxic T-lymphocyte-associated protein 4 (CTLA-4) are two ICIs that have demonstrated utility in HCC, and newer immune checkpoint targets are being tested in clinical trials. In advanced CCA and GBC, PD-1 ICIs have resulted in antitumor responses, but only in a minority of select patients. Other ICIs are being investigated for patients with CCA and GBC. Adoptive transfer may hold promise, with reports of complete durable regression in metastatic CCA, yet this therapeutic approach may not be generalizable. Alternative approaches have been developed and promising results have been observed, but clinical trials are needed to validate their utility. While the treatment of hepatobiliary cancers involves unique challenges that these cancers present, the progress seen with ICIs and adoptive transfer has solidified immunotherapy as an important approach in these challenging patients with few other effective treatment options.

## 1. Introduction

The development of novel immunotherapies has revolutionized the treatment of patients with cancer. Unlike cytotoxic chemotherapy, which functions to directly kill cancer cells or induce apoptosis, immunotherapy functions to bolster the endogenous antitumor properties of the immune system, enabling immune cells to lyse cancer cells that would otherwise escape immune surveillance. Immunotherapy comes in various forms and more novel approaches are being developed. Current therapies being used clinically include immune checkpoint inhibitors, the adoptive transfer of tumor-specific T cells including tumor infiltrating lymphocytes (TIL) or gene-engineered T cells (T cell receptor or Chimeric Antigen Receptor transduced), bispecific antibodies, vaccines, and oncolytic viruses. Immunotherapy has become a valuable treatment in many cancers, including malignant melanoma, mismatch repair-deficient colorectal cancer, human papilloma virus-positive cervical cancer, and small cell lung cancer [1,2].

One area of ongoing research is to develop and validate novel immunotherapies for the treatment of patients with hepatobiliary cancers, which includes hepatocellular carcinoma, cholangiocarcinoma, and gallbladder carcinoma. Due to the specific challenges that these cancers present, the development of new treatments has lagged behind that of other cancers [3,4]. Hepatobiliary cancers progress rapidly and are associated with a high case fatality. Since many existing treatments have limited efficacy, there is a substantial unmet clinical need to develop novel immunotherapies [5,6]. Herein, we review the current state of treatment and how immunotherapy is being integrated towards the care of patients with hepatobiliary cancers, discuss the tumor microenvironment and unique challenges to using immunotherapy in hepatobiliary cancers, and examine early-phase, novel approaches that hold great promise in treating patients with hepatobiliary cancers.

## 2. Hepatocellular Carcinoma

### 2.1. Current Therapeutic Approach

Hepatocellular carcinoma (HCC) is a primary tumor of the liver, often arising from cirrhosis, and is frequently associated with chronic hepatitis B infection, chronic hepatitis C infection, and alcoholism [7,8]. HCC is the most common primary liver tumor, making up 80–90% of all primary liver neoplasms. Approximately 900,000 new cases of liver cancer were reported in 2020 and liver cancer was the third leading cause of cancer-related deaths worldwide in the same year [9]. In the United States, liver cancer has a 5-year survival rate of 18%, making it the second deadliest site of cancer [6,10]. 

Treatment of HCC is dictated by the Child–Pugh class and/or the Barcelona Clinic Liver Cancer staging system (BCLC). Early-stage HCC that is confined to the liver is primarily treated with surgical resection and/or ablation, which seek to completely remove the tumor [11]. Transplantation is another therapeutic option that removes both the HCC as well as the underlying cirrhotic liver [6,12,13]. Despite the curative intent of these treatment modalities, tumors frequently recur and the 5-year recurrence after surgical resection can be as high as 40–70%, and recurrence after transplantation can be as high as 20–40% [6,14]. In addition, since many patients with HCC present with advanced disease or complications related to cirrhosis, only 5–10% of HCC patients are candidates for surgical therapy [15]. Transarterial therapies including transarterial chemoembolization (TACE) and selective internal radiation therapy (SIRT) can be considered for patients with unresectable, intermediate-staged BCLC B HCC [6]. While these approaches can be helpful, their efficacy as a monotherapy has been limited. 

Effective systemic therapies could potentially prolong the survival of patients with HCC. HCC, however, has been traditionally resistant to conventional cytotoxic chemotherapy, and only recently have small-molecule inhibitors been used. In 2008, sorafenib was tested in a phase III trial in patients with advanced HCC and was found to be associated with both an improvement in survival and delay in radiologic progression of 3 months versus placebo [16,17]. As a result, sorafenib was the first systemic therapy approved for the treatment of HCC. Other small-molecule inhibitors including lenvatinib, regorafenib, cabozantinib, and ramucirumab have been developed for HCC [6,18,19,20,21]. Of the newly approved molecules, only sorafenib, lenvatinib, and regorafenib have been associated with an increased median overall survival to 6.5–13.6 months depending on the study [15,16,17,18,19]. While this is an incremental improvement, the median survival for patients on these therapies remains rather dismal, highlighting the need for more efficacious systemic therapies. Given the success of immunotherapy in the treatment of some cancers, more recent efforts have been aimed at translating this success to patients with HCC.

### 2.2. The Challenging Tumor Microenvironment of HCC

The development and testing of immunotherapies among patients with HCC have taken significantly longer than in other cancers such as melanoma, mismatch repair-deficient colon cancer, or even lung cancer. While the reason for this is multifactorial, it likely is related to the reduced immunogenicity of HCC tumors. Compared with other types of tumors, such as melanoma, the tumor mutational burden of HCC tends to be low to moderate. With fewer somatic mutations within the tumor, there are a reduced number of tumor-specific neoantigens available to drive an adaptive immune response [22]. Tumor mutational burden has been used as a biomarker to indicate the efficacy of T cells against a tumor, with a lower tumor mutational burden indicating a weaker immune response [23]. In one study of 33 patients with HCC, the median TMB was 5.48 (range 1.68–16.07) and did not correlate with pathologic features of HCC [24]. More so, the impact of the tumor mutational burden in HCC on the adaptive immune response, and its relation to tumor progression and patient outcome, is poorly understood [3]. Taken together, these unique aspects to HCC have made it much more difficult to treat with immunotherapy and, importantly, have resulted in a drastic delay in the development and use of immunotherapy for patients with HCC.

Furthermore, the liver naturally creates an immunosuppressive microenvironment that facilitates tumor development. The liver provides an “immune-tolerant” environment due to its need to be accepting of new antigens encountered from food and microbial antigens delivered from the gastrointestinal tract [25]. Tolerance of antigens is partially achieved through myeloid-derived suppressor cells that secrete chemokines, cytokines, and growth factors, which increase proliferation, protect from apoptosis, increase angiogenesis, and support growth [26]. In addition, the activation and accumulation of regulatory T cells alongside upregulated programmed cell death protein 1 ligand (PD-L1) may contribute to the immunosuppressive environment. Lastly, these anti-inflammatory mediators can be increased in patients with cirrhosis [27,28,29], further contributing to the immunosuppressive tumor microenvironment of the liver. Taken together, the reduced mutational burden of primary HCC and the immunosuppressive tumor microenvironment have created a hostile landscape for the development and testing of novel immunotherapies for the treatment of HCC.

### 2.3. Immune Checkpoint Inhibitors 

**Figure 1:**
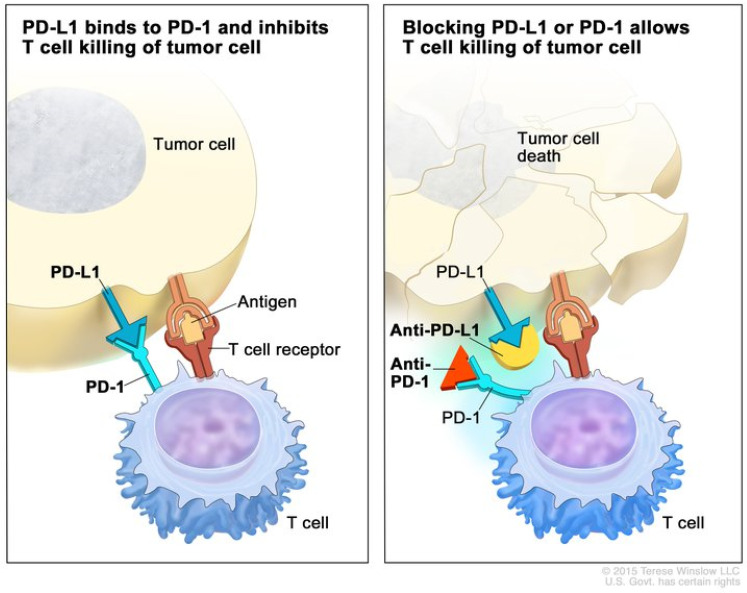
Mechanism of PD-1 and PD-L1 immune checkpoint inhibitors. (**Left**) The binding of PD-1 on the T cell to PD-L1 on the tumor cell inhibits effector T cell function, despite binding of the T cell receptor to an antigen. (**Right**) Blockade of PD-1 or PD-L1 with monoclonal antibodies prevents PD-1 binding with PD-L1 and promotes effector T cell function, leading to apoptosis of the tumor cell. Used with permission from Terrese Winslow LLC.

The first immune checkpoint inhibitor (ICI) clinical trials for HCC began in 2008 [30]. These early ICIs targeted cytotoxic T-lymphocyte-associated antigen 4 (CTLA-4). It was discovered that CTLA-4 altered intracellular T cell signaling and blocked CD28 binding to CD80 and CD86, which is needed for optimal T cell activation [31,32,33]. As a result, CTLA-4 decreases helper T cell activity and increases regulatory T cell activity [34], which further inhibits the adaptive immune response to cancer. Blocking CTLA-4 with antibodies in mouse models allowed for improved antitumor activity [35,36]. Clinical trials conducted in 2009 demonstrated that blocking CTLA-4 with monoclonal antibodies improved survival in the treatment of patients with malignant melanoma [37]. Eventually, the effects of CTLA-4 were studied in other cancers, including HCC. A phase II clinical trial of tremelimumab, an anti-CTLA-4 monoclonal antibody, in patients with hepatitis C-induced, advanced HCC achieved a partial response or stable disease in 76.4% of patients and had the added benefit of reducing hepatitis C viral load [30]. Despite these findings, the survival of patients treated with tremilimumab was only 43% at one year. While these early efforts confirmed that immunotherapy could be used to treat patients with HCC, subsequent clinical trials with more novel checkpoint inhibitors would help to solidify the role of immunotherapy as a treatment for HCC. Soon after the discovery of CTLA-4, another immune checkpoint inhibitor was discovered. The binding of the T cell surface receptor, programmed death receptor-1 (PD-1), to its ligand located on tumor cells, called programmed death ligand 1 (PD-L1), was found to inhibit T cell-mediated cytotoxicity when bound to tumor cells [34]. Early studies suggested that blockade of this interaction would improve T cell-induced immunity against cancer by disinhibiting the T cells and facilitating improved cytotoxicity [38,39,40]. Figure 1 illustrates the mechanism of anti-PD-1 therapy. Clinical trials in patients with melanoma, colorectal cancer, prostate cancer, non-small-cell lung cancer, and renal cell carcinoma established the benefit of the PD-1/PD-L1 blockade in humans and have revolutionized the treatment of patients with these cancers [41]. 

**Figure 2:**
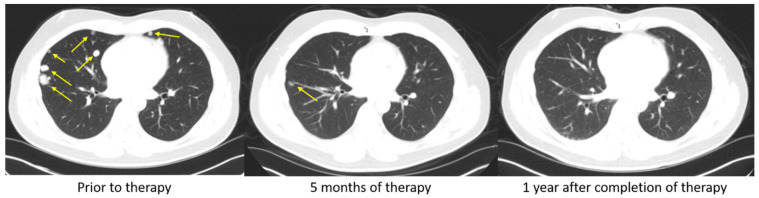
Complete response to PD-1 immune checkpoint inhibitors. A 48-year-old man with HBV cirrhosis developed a liver mass that was biopsied and found to be HCC. He underwent a laparoscopic partial right hepatectomy. Pathology revealed a poorly differentiated HCC, 3.3 cm in size, and margins were negative. On surveillance, he was found to have developed a rising AFP and to have bilateral lung nodules (Figure), but there was no evidence of recurrent or metastatic disease in the abdomen or pelvis. Biopsy of one of the pulmonary nodules confirmed metastatic disease and he was started on sorafenib. However, his disease progressed. He had no FGFR alteration, so he was started on nivolumab. After only 5 months of therapy, an objective response was seen on CT scan (middle). He completed a full year of therapy, and he remains without evidence of disease one year after completion of therapy.

Studies of the immune landscape within the TME of HCC have found that exhausted CD8+ T cells (those that overexpress PD-1) were both preferentially enriched and potentially clonally expanded. As such, the PD-1/PDL-1 axis represents an important pathway to target in order to induce an immune response in patients with HCC [42]. In 2016 and 2017, anti-PD1/PD-L1 monoclonal antibodies were studied in advanced, unresectable HCC. In two phase II trials, nivolumab and pembrolizumab prolonged survival and resulted in a 15–20% objective response rate (ORR), of which there was one (1%) complete and 17 (16%) partial responses in the KEYNOTE-224 [43,44]. These findings were confirmed in two additional phase III clinical trials. In the study by Finn et al., second-line therapy with pembrolizumab following treatment with sorafenib resulted in reduced progression in advanced HCC over two years versus placebo. The study also demonstrated prolonged survival; however, despite the results being significantly different from placebo, the prolonged survival failed to meet the statistical threshold set by the investigators [3,45]. Likewise, a phase III trial comparing nivolumab to sorafenib in patients with advanced HCC resulted in an improvement in both 1-year and 2-year survival. However, with a minimum follow-up of 22.8 months, overall survival was not significantly different between the two cohorts [46]. In contrast, longer-term studies with a minimum follow-up of 33.6 months resulted in improved safety and survival among patients treated with nivolumab compared with sorafenib when used in the setting of advanced HCC [47]. Figure 2 demonstrates a patient with advanced HCC that progressed on sorafenib and subsequently achieved a complete response after one year on nivolumab. In another phase III clinical trial, Finn et al. studied the effects of Atezolizumab, an anti-PD-L1 monoclonal antibody, in combination with Bevacizumab, a vascular endothelial growth factor (VEGF) inhibitor, in advanced HCC. The combination of Atezolizumab with Bevacizumab led to an overall survival of 67.2% at 12 months and a median progression-free survival of 6.8 months. This was superior to sorafenib, which had a 12-month overall survival and median progression-free survival of 54.6% and 4.3 months, respectively. This trial demonstrates that combination therapy with Atezolizumab with Bevacizumab may be superior to monotherapy with sorafenib in patients with advanced HCC [48]. 

While these studies demonstrated a moderate improvement in outcomes with the use of specific ICIs as monotherapy compared with sorafenib, the benefits are more obvious when different ICIs are combined. Combination therapies allow targeting of multiple, upregulated immune checkpoint pathways, which may improve patient outcomes versus monotherapy with ICIs. However, these findings must be balanced with the potential for increased toxicity and immune-related adverse events. The value of targeting multiple ICIs has been demonstrated in patients with melanoma, and this strategy is under active investigation in patients with HCC [49]. One ongoing study (NCT02519348) is treating patients with HCC with durvalumab (anti-CTLA-4) and tremelimumab (anti-PD-1 agent). Early results from this phase I/II trial indicated that a single, increased dose of tremelimumab combined with a standard regimen of durvulumab led to an objective response rate (ORR) of 22.7% and a median overall survival of 18.7 months, with an acceptable side effect profile [50]. The combination of tremiliumumab and durvulumab is now being studied in an ongoing phase III clinical trial (NCT03298451) as a first-line treatment for advanced HCC in patients who are ineligible for locoregional therapy. Another study (NCT01658878) reported the safety and efficacy of nivolumab in combination with ipilimumab in patients with advanced HCC. The study included three arms with combination therapy, each with a varying dose and schedule of nivolumab and ipilimumab. The lack of comparison to monotherapy arms makes the synergistic benefits unclear; however, the investigators concluded that patients on both medications developed a 31% ORR, which included a complete response rate of 6%, partial response rate of 24%, and a tolerable safety profile [46]. Another study from the same investigator (NCT01658878) demonstrated that adding ipilimumab to a regimen of nivolumab and cabozantinib (receptor tyrosine kinase inhibitor) resulted in an improved ORR and median progression-free survival, but at the cost of increased grade 3 and 4 adverse events [51]. In addition to these trials, numerous ongoing clinical trials are being conducted to determine the therapeutic benefit of various ICI combinations [52]. Other studies from the National Cancer Institute (NCT02465060) and from the American Society of Clinical Oncology (NCT02693535) are using next-generation sequencing of paraffin-embedded tumors after resection to improve patient selection for available systemic therapies. Both studies are investigating a wide variety of cancer types, including hepatobiliary cancers. Genotyping studies such as these will determine the utility of individualized treatment and may eventually inform the use of individualized immunotherapies.

With evidence mounting to support the use of immunotherapy for advanced HCC alone or in combination with other systemic therapies, other studies have been conducted to investigate whether there is an advantage of combining ICIs with liver-directed therapies. A phase I/II study by Duffy et al. combined tremelimumab (anti-CTLA-4) with transarterial chemoembolization (TACE) or ablation and reported a median overall survival of 12.8 months. Notably, patients who received TACE instead of ablation had improved survival, with a 12-month survival rate of 80.8%. The investigators also noted an increase in activated CD4+ and CD8+ T cells in the peripheral blood, though differences in TIL between pre- and post-treatment samples were not significant. In addition, 12 out of 14 patients with concurrent hepatitis C infection had reduced viral loads. While impressive, this early study lacked control arms of patients treated with transarterial therapy alone or immunotherapy alone [53]. Additional clinical trials (NCT04517227, NCT03753659, and NCT04102098) to evaluate the efficacy of PD-1 inhibitors combined with ablation or TACE for HCC are currently accruing patients. In addition, several other clinical trials are evaluating the value of adding ICIs to other systemic therapies and small-molecule inhibitors such as vascular endothelial growth factor (VEGF) inhibitors and kinase inhibitors [52]. Such studies include NCT03713593, NCT03605706, and NCT03006926, among others, and are anticipated to complete accrual in the next 2–5 years. 

Other ICIs are in development but have yet to see widespread clinical use. Lymphocyte-activation gene 3 (LAG-3) and T-cell immunoglobulin mucin-3 (TIM-3) are being studied as potential targets for ICIs. LAG-3 binds to major histocompatibility complex (MHC) class II and is found on CD8+ T cells [54]. LAG-3 expression is increased in HCC tumor-infiltrating lymphocytes (TILs) and LAG-3 expression is correlated with impaired T cell effector function [55]. Mouse models have been used to validate the efficacy of LAG-3 blockade. In mice, anti-PD-1 and anti-LAG-3 antibodies work synergistically to induce a complete response in the majority of those bearing syngeneic melanoma or colorectal tumors. This is in contrast to only a small minority of mice that develop complete remission when treated with either agent alone [56]. Based on these preclinical results, phase I clinical trials are now open and are evaluating the safety and efficacy of LAG-3-targeted ICIs in HCC. NCT04658147 is evaluating Relatlimab, an anti-LAG-3 monoclonal antibody, among patients with HCC, while NCT03849469 is assessing the utility of a bispecific antibody (an antibody capable of binding two receptors at once) that targets both CTLA-4 and LAG-3. Table 1 contains a full list of ongoing clinical trials using a combination of immune checkpoint inhibitors and other novel immunotherapies for HCC.

TIM-3 is expressed on a variety of cells, including innate immune cells, CD4+ cells, and CD8+ cells [57,58]. Similar to LAG-3, PD-1, and CTLA-4, TIM-3 has been demonstrated to impair the adaptive immune response to cancer when bound to its cognate ligand [59]. TIM-3 is upregulated in the TILs of HCC patients, and increased TIM-3 expression is associated with decreased survival and increased recurrence [60]. Meanwhile, blocking TIM-3 binding to its ligand, galectin-9, reduces the population of regulatory T cells (T cells that inhibit the immune response to cancer), improves TIL proliferation, and improves effector cytokine production [58,61]. These studies highlight the importance of TIM-3 in the immune response to HCC. There are several ongoing phase I and phase II clinical trials that are evaluating the efficacy and safety of TIM-3 ICIs [59]. One study from the University of Hawaii (NCT03680508) is specifically studying cobolimab, an anti-TIM-3 antibody, with dostarlimab, an anti-PD-1 antibody, in 42 liver cancer patients. While promising, the efficacy of TIM-3 ICIs in humans remains unknown.

The efficacy of immune checkpoint inhibitors may be improved with the targeting of Indoleamine 2,3-dioxigenase 1 (IDO1). IDO1 is involved in the conversion of L-tryptophan into L-kynuernine and promotes immunosuppression by depleting L-tryptophan in effector cells and producing L-kynuernine derivatives that ultimately create signals leading to increased regulatory T cell differentiation [62,63]. In mouse models of HCC, IDO1 blockade along with anti-CTLA-4 treatment proved a more effective means to reduce tumor growth than anti-CTLA-4 monotherapy, paving the way for IDO1 inhibitors to be tested in combination therapies [64]. Currently, one clinical trial (NCT03695250) is investigating the safety and efficacy of an IDO1 inhibitor, BMS-986205, paired with nivolumab in patients with stage III and stage IV HCC. 

### 2.4. Future Directions and Novel Approaches

Other forms of immunotherapy beyond ICIs are currently in development and may be tested in patients with HCC. One such example is the adoptive cell transfer (ACT) of immune cells. ACT involves the collection of immune cells from a cancer patient, ex vivo enrichment and expansion or genetic modification, followed by the administration of the expanded T cells back into the patient [65]. The ACT of tumor-infiltrating lymphocytes (TIL) has proven to be an effective treatment in advanced melanoma, some types of cervical cancer, and small cell lung cancer, but its interest in HCC is still being investigated [65,66,67]. In an early randomized trial using autologous tumor-reactive peripheral blood mononuclear cells (PBMC) harvested prior to surgical resection, transfer of PBMCs led to an 18% decrease in recurrence compared with corrected control patients that only underwent surgery. However, with a median follow-up of 4.4 years, overall survival was no different between the two cohorts [68]. 

Several studies have investigated the use of another type of cellular therapy in patients with HCC called cytokine-induced killer (CIK). CIK cells are expanded ex vivo from peripheral blood mononuclear cells and function to improve the immune response to cancer through non-MHC-restricted antitumor activity. In a meta-analysis of 13 clinical trials, CIK cell infusion was noted to improve 1- and 2-year survival in patients with HCC [69]. In this study, Pan et al. identified independent predictors of overall survival in CIK immunotherapy, including tumor size, tumor capsule, pathological grades, total bilirubin, albumin, prothrombin time, alpha-fetoprotein, and tumor number [70,71]. While the results of CIK treatments for HCC have been promising, and large-scale, phase III studies have been performed, the treatment has yet to be widely implemented. 

More recently, CAR-T immunotherapy, which genetically modifies T cells using lentiviruses or retroviruses, has emerged as a promising therapy. These T cells begin to express CAR, which can bind antigens without MHC antigen presentation. Once CAR-T cells are identified, they are expanded ex vivo and these cells are adoptively transferred into the patient [72,73]. Due to the harsh tumor immune microenvironment, the use of the adoptive transfer of ex-vivo-expanded tumor reactive T cells has gained traction. By enriching for a specific population of T cells that have a known affinity for a patient’s cancer-specific antigens and injecting these cells at large numbers into the patient, researchers have tried to overcome the harsh immune-inhibitory tumor microenvironment and induce tumor regression. In xenograft models of HCC, CAR-T cells have been used successfully to inhibit tumor growth and, in some instances, eradicate tumors. However, concerns exist about the safety of CAR-T immunotherapy due to the potential for off-target toxicity, as well as their ability to effectively function in the immunosuppressive environment of the human liver [74,75,76]. The future of adoptive cell transfer for HCC will likely require more studies. Fortunately, numerous ongoing clinical trials are investigating the utility of ACT for HCC with a particular emphasis on CAR-T immunotherapy. 

Inducing an immune response to cancer through the administration of tumor surface antigens that are shared between tumors and patients is another approach currently being investigated. Tumor peptides can be packaged or delivered in various ways to optimize the immune response. Cancer vaccines are based on the delivery of full-length tumor antigens or corresponding epitope peptides. These antigenic sequences can be delivered either as naked peptides/proteins, naked DNA/RNA, or vectored via recombinant viruses (oncolytic or not) or even bacteria, or via loaded cells such as dendritic cells. One phase I clinical trial in HCC patients comparing dendritic cell infusion, dendritic cell infusion with sorafenib, and sorafenib alone noted that dendritic cell infusion led to increased tumor-specific CD8+ populations in peripheral blood, without evidence of autoimmune reactions, and with a disease control rate of 35%. Overall survival was difficult to assess as median overall survival was much lower than that in historical controls [77]. 

Traditional peptide vaccines have also been studied. A glypican-3-derived peptide vaccine was well tolerated in a phase I trial. Out of 33 patients treated, only one partial response was observed; nineteen patients had stable disease and the remaining had progressive disease. The median overall survival in this study was 9 months [78]. Another peptide vaccine, this time with a telomerase peptide, did not exhibit antitumor efficacy in a phase II clinical trial of HCC patients [79]. Numerous other peptide vaccine candidates are being studied in HCC, including alpha-fetoprotein, NY-ESO, SSX-2, and melanoma antigen-encoding gene A [80]. Peptides can also be packaged into viruses that are designed to infect rapidly dividing cancer cells. After tumor cell infection, viral RNAs are expressed and encode for antigenic peptide or protein sequences, thus aiding in stimulating an immune response to cancer. This exciting approach has been tested clinically in patients with HCC. Pexastimogene devacirepvec (Pexa-Vec), an oncolytic vaccine that preferentially infects HCC cancer cells, increased overall survival, particularly when administered at high doses (109 PFU), in a phase II clinical trial [81]. A phase III clinical trial, NCT02562755, evaluating the efficacy of Pexa-Vec, was completed in December 2020 and is awaiting published results. The results of these promising studies may determine the direction of novel immunotherapies in HCC.

While progress is being made and immunotherapy is proving itself to be an important approach in the armamentarium of the treating oncologist, significant challenges remain. Unlike other tumors, such as pancreatic cancer, colon cancer, or malignant melanoma, there is no reliable biomarker available to predict tumor response to immunotherapy in patients with HCC [82,83]. In addition, the fact that neither tumor mutational burden nor the expression of PDL-1 is predictive of the tumor response to ICIs in patients with HCC makes patient selection for immunotherapy difficult (Oncotarget 2019, 10, 4018–4025; Genome Med. 2019, 11, 28). Despite these challenges, novel clinical trials and continued efforts will ultimately decide the continued role of immunotherapy in patients with HCC.

## 3. Biliary Tract Cancer

### 3.1. Current Therapeutic Approach

Cholangiocarcinoma (CCA) and gallbladder carcinoma (GBC) are both malignancies that can arise from the biliary tract. CCA is a neoplasm of the bile duct, generally originating from cholangiocytes, which can be located in the intrahepatic (arising proximal to the formation of the common hepatic duct), perihilar (proximal to common bile duct), or distal (distal to common bile duct) biliary tree [84]. Risk factors, clinical presentation, and prognosis vary depending on CCA location [84]. The incidence of CCA has been increasing and varies based on geography [84,85]. The frequency of GBC also varies by geographic region and most new cases are identified incidentally during routine cholecystectomy [86,87,88]. Cancers of the gallbladder are relatively rare, yet advanced stages of gladder cancer can be associated with a high case fatality [9]. In fact, patients with advanced GBC can have 5-year survival in the range of 5%; however, earlier stages of disease can have a much better prognosis, with 5-year survival in the range of 75–85% [86,87,89].

Biliary tract cancers are generally characterized by an aggressive tumor biology and most patients present at advanced stages that preclude surgical resection. Transplantation may be an option for select patients with unresectable hilar CCA [84,90,91,92,93,94]. For patients with GBC, resection typically involves removal of the gallbladder segment 4b/5 with a concomitant lymphadenectomy, as well as bile duct resection depending on the margin status of the cystic duct [89]. Despite curative-intent surgery, recurrence can occur within the first two years of resection and 5-year disease-free survival can be as low as 20% depending on disease stage [84,95,96,97,98,99,100,101]. Similarly, the median overall survival of patients with distal, perihilar, and intrahepatic CCA after surgical resection has been relatively poor, at 21.9 months, 35–40 months, and 18–39 months, respectively [102,103,104,105]. 

For patients with locally advanced or metastatic disease, the administration of systemic, cytotoxic chemotherapy remains the mainstay of therapy. First-line treatment generally consists of gemcitabine with cisplatin, which has been demonstrated to be superior to gemcitabine monotherapy [106]. Second-line therapies consist of various combinations of chemotherapy and/or small-molecule inhibitors including VEGF inhibitors, IDH1 inhibitors, or FGFR2 inhibitors [107]. Despite the numerous combinations and targeted agents available, the most efficacious second-line therapy for biliary tract cancer has been difficult to determine. Triple combination therapy with folinic acid, 5-FU, and oxaliplatin may be a more promising regimen, but, overall, outcomes remain dismal. As such, there is a significant clinical need to develop novel approaches to treat biliary tract cancer. Given recent successes using immunotherapy for patients with solid tumors that have historically been considered “immune-cold”, there has been increasing interest in the medical community to test immunotherapy in patients with biliary tract cancers [4,84].

### 3.2. The Challenging Tumor Microenvironment of Biliary Tract Cancers

Biliary tract tumors are characterized by genomic heterogeneity. In addition to arising in varied locations throughout the biliary tract and gallbladder, each tumor has a unique tumor microenvironment with significant variation at both the genomic and epigenetic levels within cancer cells [108]. GBC often arises from chronic inflammation of the gallbladder, including gallstones, porcelain gallbladder, chronic cholecystitis, and primary sclerosing cholangitis [86,87,109,110,111]. Meanwhile, the main etiology of CCA in western countries is primary sclerosing cholangitis (PSC). CCA often arises sporadically, though several conditions, including parasitic infections, biliary tract disorders such as PSC, and exposure to toxins, have been identified as risk factors for CCA [112]. In turn, the tumor microenvironment in CCA can be characterized by chronic inflammation and excessive cytokine secretion. Of note, IL-6 may induce proliferation in neoplastic cholangiocytes and epigenetic changes induce a constitutive state of IL-6/STAT-3 signaling [113,114]. Tumor-associated-macrophages infiltrate a majority of CCA tumors in high densities. These macrophages are associated with poorer survival and may have a role in supporting metastasis and degrading the extracellular matrix [115]. In addition, studies in other cancers have demonstrated that tumor-associated-macrophages may aid in the immune escape of cancer cells and immunosuppression by IL-10 secretion [116]. Similarly, the microenvironment in GBC is characterized by sustained inflammatory signaling molecules, which creates a hostile environment for the host adaptive immune response to cancer [117].

Several biomarkers are being studied as predictors of response to therapy and to define a cohort of patients more likely to benefit from immunotherapy. Immunohistochemistry staining of CCA tumors has demonstrated that 7.3% of intrahepatic CCA and 5.2% of perihilar or distal CCA are PD-L1 positive, indicating that PD-L1 is relatively poorly expressed in CCA compared with other cancers such as melanoma or colon cancer. The minority of tumors that are PD-L1 positive also express higher rates of other biomarkers including BRAF, BRCA2, RNF43, TP53, and TOP2A mutations, with increased tumor mutational burden and increased microsatellite instability, which could lead to more therapeutic options for patients with PD-L1-positive tumors [118]. In addition, regulatory T cells in CCA tend to express high levels of CTLA-4 and FoxP3, contributing to immune escape [119,120]. In GBC, cancer cells with ectopic expression of ERBB2/ERBB3 mutants had increased PD-L1 expression, indicating the potential advantages of PD-L1 blockade in these patients [121]. Taken together, these biomarkers may be used to better select patients for immunotherapy.

### 3.3. Immune Checkpoint Inhibitors

As with HCC, PD-1 and CTLA-4 ICIs are currently the most studied forms of immunotherapy in patients with biliary tract cancers. Unfortunately, anti-PD-1 ICIs have not yet demonstrated robust utility for CCA and GBC. In a phase II study (NCT02628067) and in a phase Ib study (NCT02054806) of pembrolizumab in advanced biliary tract cancer, durable antitumor activity was only noted among 6–13% of patients [122]. Similarly, a phase II trial (NCT02829918) of nivolumab for advanced, refractory biliary tract cancer noted that nivolumab led to a modest ORR of 11%, including one partial response, and a disease control rate of 50%. Interestingly, all responders to nivolumab in this trial had mismatch repair protein-proficient tumors [123]. These data contrast with other studies in which mismatch repair status was a predictor of response to pembrolizumab [124,125]. The disparate results may be due to the rarity of mismatch repair-impaired or microsatellite instability-high CCA [125,126]. In a phase II clinical trial (NCT02628067) of several non-colorectal cancers, including 22 CCA patients, the ORR of pembrolizumab for tumors with impaired DNA mismatch repair and with high microsatellite instability was 34.3%. This study was, however, difficult to apply specifically to biliary tract cancers since it also included multiple other forms of cancer, yet it did not include GBC. The findings could, however, indicate that DNA mismatch repair and microsatellite instability biomarkers still predict the response to PD-1 blockade despite their rarity [127]. These studies demonstrates that, while PD-1 blockade may be beneficial in the treatment of biliary tract cancers, this benefit may not be universal and may be predicted using biomarkers to individualize therapy. Given the rarity of traditional biomarkers used to predict the success of anti-PD-1 therapy, novel biomarkers or ex vivo models to predict patient responses to ICIs are an area of active research. In addition, other clinical trials are ongoing to characterize anti-PD-1 ICI for biliary tract cancers, including several phase III trials (NCT04003636 and NCT04924062). 

Compared with PD-1, the benefit of CTLA-4 blockade for biliary tract cancers is poorly understood. Interestingly, immunohistochemistry of paraffin-embedded tumor blocks from perihilar and distal CCA patients who underwent surgery demonstrated that high levels of CTLA-4 on TILs were associated with prolonged overall survival, suggesting that CTLA-4 may have prognostic value [128]. In general, TILs in CCA overexpress CTLA-4 and ex vivo blockade of CTLA-4 in CCA-derived TILs can lead to increased effector T cell function and proliferation [129]. The majority of clinical trials with CTLA-4 ICIs focus on combination therapy. Preliminary results from a recent phase I clinical trial (NCT01938612) noted that durvalumab with tremelimumab for biliary tract cancer led to a median overall survival of 10.1 months versus 8.1 months with durvalumab monotherapy [130]. Another phase II trial (NCT03046862) studied the same drug combination yet in combination with gemcitabine and cisplatin for advanced biliary tract cancer. The ORR, disease control rate, and the median overall survival in the durvalumab plus chemotherapy group versus the durvalumab plus tremelimumab group were 73.4% vs. 73.3%, 100% versus 97.8%, and 18.1 months vs 20.7 months, respectively [131]. The similarities in outcomes between these two arms suggests that the addition of multiple ICIs to chemotherapy was not more effective than a single ICI with chemotherapy. 

Ongoing trials with CTLA-4 ICIs continue to focus on combination therapies. A phase II clinical trial, NCT02834013, is investigating nivolumab and ipilimumab in rare tumors including CCA and GBC. Similarly, another phase II study (NCT04634058) is investigating combination ICIs, but with anti-PD-L1 antibodies instead of anti-PD-1 in combination with CTLA-4 antibodies, for patients with advanced intrahepatic cholangiocarcinoma following progression despite receiving standard chemotherapy. Other studies are investigating combination ICIs alongside novel chemotherapies (NCT03058289) or with loco-regional therapies (NCT02821754). In addition, NCT03849469, which was previously discussed relative to HCC, is also investigating a bispecific antibody for CTLA-4 and LAG-3 for intrahepatic cholangiocarcinoma. A list of ongoing clinical trials with combination immune checkpoint inhibitors and novel immunotherapies in CCA or GBC can be found in Table 2 and Table 3.

Few studies have investigated other ICI targets besides PD-1 and, to a lesser degree, CTLA-4. It has been observed that 45% of biliary tract tumors express high levels of immune checkpoint inhibitors such as IDO-1, LAG-3, HAVCR2, TNFRSF9, BTLA, CD274, PDCD1, and TNFRSF4. In addition, increased activity of immune checkpoint molecules has been associated with worse prognosis [132]. There are few ongoing clinical trials studying these targets of ICIs. One phase Ib clinical trial (NCT04641871) is evaluating anti-PD-1 therapies in combination with either anti-LAG-3 or anti-TIM-3 in a variety of metastatic cancers, including BTC. More studies are needed to determine whether immune checkpoint targets may improve CCA treatments. 

### 3.4. Future Directions and Novel Approaches

Other immunotherapies are in development for CCA and GBC, including ACT and tumor vaccines. Due to the harsh tumor immune microenvironment in patients with GBC, the use of the adoptive transfer of ex-vivo-expanded tumor-reactive T cells has gained traction. By enriching for a specific population of T cells that have a known affinity for a patient’s cancer cells and injecting these cells at large numbers into the patient, researchers have tried to overcome the harsh immune-inhibitory tumor microenvironment. Success using ACT for CCA has been described in case studies, but it has not yet been confirmed in larger trials [133,134]. The potential of ACT using fourth-generation CAR-T cells targeting CD133 has been demonstrated in ex vivo tissue models [135]. CD133 is an attractive target since it is expressed in over 50% of biliary cancers. Another phase I clinical trial (NCT01869166) studied CAR-T cells in EGFR-positive, advanced, unresectable biliary cancers. The investigators concluded that CAR-T cell therapy was safe in this population and, in the 17 evaluable patients from the study, one patient achieved a complete response and 10 patients achieved stable disease [136]. Additionally, a phase I/II clinical trial (NCT04426669) is studying ACT in gastrointestinal tumors, including GBC, using TIL after knockdown of cytokine-induced SH2 protein by CRISPR gene editing. ACT with CIK cells is another area of ongoing research. One phase I/II study in Thailand (NCT01868490) is using ACT of CIK cells in CCA. There is also a phase III trial (NCT02482454) using ACT of CIK cells in conjunction with radiofrequency ablation for CCA. Given the rarity of GBC, the anticipated completion of accrual is estimated to occur in 2030, and this highlights another challenge in the development of novel therapies for such a rare disease.

Early studies have been performed using a variety of tumor vaccines for biliary tract cancers. Tumor vaccines are an attractive approach to biliary tract cancers due to the rapidly dividing nature of BTCs and the unique, macrophage-rich tumor microenvironment that the liver provides. Peptide vaccines have been designed to induce an immune response to the cell surface molecules Wilms Tumor 1 (WT1) and Mucin 1 (MUC1). In one study using a WT1 peptide vaccine and gemcitabine combination therapy for pancreatic and biliary tract cancers, including eight patients with advanced CCA and eight with advanced GBC, the disease control rate in biliary tract cancer was 50% and median overall survival was 278 days [137]. A phase I study of the MUC1 vaccine in advanced pancreatic and biliary tract cancers, which included three patients with biliary tract cancer, concluded that the vaccine was safe. However, two of the three patients with biliary tract cancer continued to have progressive disease and the other was not evaluated, making it difficult to determine the benefit of this treatment [138]. 

Improved response rates have been observed using vaccines in patients with biliary tract cancers when multiple, different peptides were combined in the vaccine treatment. In a phase I clinical trial Aruga et al. demonstrated that peptide vaccines for cell division cycle-associated 1 (CDCA1), cadherin 3 (CDH3), and kinesin family member 20A achieved stable disease in five out of nine patients and a median overall survival of 9.7 months [139]. In a separate study, Aruga et al. tested a different vaccine with four peptides (lymphocyte antigen 6 complex locus K, TTK protein kinase, insulin-like growth factor-II mRNA-binding protein 3, and DEP domain containing 1) and observed a median overall survival of 380 days [140]. A phase I/II clinical trial (UMIN000005820) studied the utility of dendritic cell vaccines in combination with ACT of CD3 activated peripheral blood T cells in patients undergoing surgical resection for intrahepatic CCA. This combination of immunotherapy improved median overall survival from 17.4 months in the surgery only group to 31.9 months in the surgery plus immunotherapy group [141]. Several ongoing studies are using peptide vaccines (NCT04853017) or autologous dendritic cell vaccines (NCT03942328). While several proof of concept studies have been performed, a significant improvement in objective response rate and survival will need to be achieved to support the use of tumor vaccines in biliary tract cancers. 

While oncolytic viruses have not been used clinically, there is increasing preclinical evidence to support the efficacy of this approach in patients with GBC or CCA. For example, the adenovirus AxdAdB-3 reduced the growth of subcutaneous GBC tumors in nude mice compared to placebo, and the addition of 5-Fluorouracil to the viral treatment resulted in complete tumor regression in almost 50% of mice treated [142]. Other oncolytic viruses, including a replication-competent herpes simplex virus, as well as a myxoma virus, have shown preclinical efficacy in both in vitro and in vivo preclinical models of GBC [143,144]. Similarly, oncolytic viruses have been used successfully in preclinical models of CCA. In a study by Pugalenthi et al., the vaccinia virus, GLV-1h68, was able to infect, replicate, and lyse three human CCA cell lines in vitro. In addition, when a single dose of GLV-1h68 was given as an intratumoral injection to nude mice bearing CCA flank tumors, a 46% reduction in tumor volume was observed compared to those injected with phosphate-buffered saline (PBS) [145]. Given the promise of these approaches, significant efforts are being undertaken to translate these results in human clinical trials.

## 4. Conclusions

The aggressive tumor biology, reduced tumor mutational burden, and immunosuppressive tumor microenvironment characteristic of hepatobiliary cancers have significantly delayed the development and adoption of novel immunotherapies for the treatment of patients with hepatocellular carcinoma (HCC), cholangiocarcinoma (CCA), and gallbladder cancer (GBC). However, the once barren therapeutic landscape has begun to change in recent years, partly because of significant breakthroughs using immune checkpoint inhibitors (ICIs). ICIs are now standard of care in patients with unresectable or metastatic HCC, and new immune checkpoints are being investigated alone and in combination with established other therapies. Similar to HCC, recent prospective clinical trials have established immunotherapy as a valuable treatment option in select patients with CCA and GBC, but overall objective response rates are much lower and overall survival remains poor. Immunotherapy represents a potential avenue for developing new treatments, but research on other immune checkpoint inhibitors and other forms of immunotherapy is rare in patients with CCA and GBC. Additional research is needed to evaluate further the utility of immunotherapy. While other approaches including cellular therapies, oncolytic viruses, and tumor vaccines are on the horizon and may prove beneficial for patients with hepatobiliary cancers, special consideration will be needed to avoid autoimmunity and toxicity in this often frail patient population. 

## Figures and Tables

**Table 1:** Clinical trials currently evaluating immunotherapies in HCC. List of clinical trials involving combination immune checkpoint inhibitors, adoptive cell transfer, and other innovative immunotherapies in HCC. Source: clinicaltrials.gov (28 July 2021). HCC, hepatocellular carcinoma; TIL, tumor infiltrating lymphocyte; CAR-T cell, chimeric antigen receptor T cell.

Trial Identifier	Phase	Status	Immunotherapy	Co-Treatments	Participants
NCT04740307	II	Recruiting	Combination immune checkpoint inhibitors (CTLA-4 with PD-1)	Lenvatinib	110
NCT02821754	II	Recruiting	Combination immune checkpoint inhibitors (CTLA-4 with PD-1)	Transarterial catheter chemoembolization, radiofrequency ablation, cryoablation	90
NCT04785287	I/II	Recruiting	Combination immune checkpoint inhibitors (CTLA-4 with PD-1)	Stereotactic Body Radiation Therapy	80
NCT04430452	II	Not yet recruiting	Combination immune checkpoint inhibitors (CTLA-4 with PD-1)	Hypofractionated Radiation Therapy	30
NCT03638141	II	Recruiting	Combination immune checkpoint inhibitors (CTLA-4 with PD-1)	Drug-eluting bead transarterial chemoembolization	30
NCT03222076	II	Active not recruiting	Combination immune checkpoint inhibitors (CTLA-4 with PD-1)	N/A	30
NCT03652077	I	Active, not recruiting	Immune checkpoint inhibitor (TIM-3)	N/A	40
NCT03680508	II	Recruiting	Combination immune checkpoint inhibitors (TIM-3 with PD-1)	N/A	42
NCT04658147	I	Recruiting	Combination immune checkpoint inhibitors (LAG-3 with PD-1)	N/A	20
NCT03695250	I/II	Active, not recruiting	Immune checkpoint inhibitor (PD-1) with IDO1 inhibitor	N/A	8
NCT04728321	II	Recruiting	Bispecific antibody against CTLA-4 and PD-1	lenvatinib	75
NCT04444167	I/II	Recruiting	Bispecific antibody against CTLA-4 and PD-1	Lenvatinib	30
NCT04601610	I/II	Not yet recruiting	Bispecific antibody against CTLA-4 and PD-L1	Ningetinib Tosylate (tyrosine kinase inhibitor)	70
NCT03849469	I	Recruiting	Bispecific antibody against CTLA-4 and LAG-3, combination bispecific antibody and immune checkpoint inhibitor (PD-1)	N/A	242
NCT03980288	I	Recruiting	GPC3 CAR-T cell	Fludarabine, cyclophosphamide	36
NCT04121273	I	Recruiting	GPC3 CAR-T cell	N/A	20
NCT03884751	I	Recruiting	GPC3 CAR-T cells	N/A	15
NCT02905188	I	Recruiting	GPC3 CAR-T cells	Cytoxan, Fludarabine	14
NCT04951141	I	Recruiting	GPC3 CAR-T cells	N/A	10
NCT03302403	N/A	Active, not recruiting	GPC3 CAR-T cell	Fludarabine, Cyclophosphamide	18
NCT04506983	1	Not yet recruiting	GPC3-CAR-T cells	N/A	12
NCT03198546	I	Recruiting	GPC3 and/or TGFβ targeting CAR-T cells	N/A	30
NCT03638206	I/II	Recruiting	DR5 CAR-T cells	N/A	73
NCT03941626	I/II	Recruiting	DR5 CAR-T/TCR-T cells	N/A	50
NCT03993743	I	Recruiting	CD147 CAR-T cells	N/A	34
NCT04550663	I	Not yet recruiting	NKG2dLs CAR-T cells	N/A	10
NCT03441100	1	Recruiting	MAGEA1 TCR engineered T cells	Cyclophosphamide, fludarabine, Interleukin-2	15
NCT04502082	I/II	Recruiting	Alpha fetoprotein peptide/HLA-A2 complex TCR engineered T cells	N/A	50
NCT04634357	I/II	Not yet recruiting	Alpha fetoprotein peptide/HLA-A2 complex TCR engineered T cells	N/A	25
NCT04518774	I	Recruiting	expanded allogeneic gamma-delta T cells	N/A	8
NCT03836352	II	Recruiting	Induction of survivin-specific cytotoxic T lymphocytes with immune checkpoint inhibitors (PD-1)	Cyclophosphamide	184
NCT04417764	I	Recruiting	PD-1 knockout engineered T cells	Transarterial catheter chemoembolization	10
NCT04317248	II	Recruiting	Autologous dendritic cell vaccine	Cyclophosphamide	600
NCT04912765	II	Recruiting	Neoantigen Dendritic Cell Vaccine with immune checkpoint inhibitor (PD-1)	N/A	60
NCT04147078	I	Recruiting	Autologous dendritic cells vaccine	N/A	80
NCT03228667	II	Active, not recruiting	PD-L1 targeting high-affinity natural killer with immune checkpoint inhibitors (PD-1 or PD-L1)	N-803 (interleukin-15 superagonist)	145
NCT03311334	I/II	Recruiting	Peptide vaccine against WT1 with immune checkpoint inhibitors (PD-1)	N/A	104
NCT04246671	I/II	Recruiting	Peptide vaccine against HER-2/neu	N/A	45
NCT02432963	I	Active, not recruiting	Peptide vaccine against P53 with immune checkpoint inhibitors (PD-1)	N/A	19
NCT04251117	I/II	Recruiting	Personalized neoantigen DNA vaccine with immune checkpoint inhibitor (PD-1)	Plasmid-encoded interleukin 12	24
NCT04248569	I	Recruiting	Peptide vaccine against DNAJB1-PRKACA fusion kinase with combination immune checkpoint inhibitors (CTLA-4 with PD-1)	N/A	12
NCT03071094	I/II	Active, not recruiting	Oncolytic vaccine with immune checkpoint inhibitor (PD-1)	N/A	30
NCT04665362	I	Not yet recruiting	Oncolytic vaccine with immune checkpoint inhibitor (PD-1)	Apatinib	10
NCT04665362	I	Not yet recruiting	Oncolytic virus with immune checkpoint inhibitor (PD-1)	Apatinib	10

**Table 2:** Clinical trials currently evaluating immunotherapies in CCA. List of clinical trials involving combination immune checkpoint inhibitors, adoptive cell transfer, and other innovative immunotherapies in CCA. Source: clinicaltrials.gov (28 July 2021). CCA, cholangiocarcinoma; TIL, tumor-infiltrating lymphocyte; CAR-T cell, chimeric antigen receptor T cell; CIK, cytokine-induced killer.

Trial Identifier	Phase	Status	Immunotherapy	Co-Treatments	Participants
NCT02834013	II	Recruiting	Combination immune checkpoint inhibitors (CTLA-4 with PD-1)	N/A	818
NCT03473574	II	Active, not recruiting	Combination immune checkpoint inhibitors (CTLA-4 with PD-1)	Gemcitabine, Cisplatin	128
NCT02821754	II	Recruiting	Combination immune checkpoint inhibitors (CTLA-4 with PD-1)	Transarterial catheter chemoembolization, radiofrequency ablation, cryoablation	90
NCT04634058	II	Not yet recruiting	Combination immune checkpoint inhibitors (CTLA-4 with PD-1)	N/A	40
NCT03058289	I/II	Recruiting	Combination immune checkpoint inhibitors (CTLA-4 with PD-1)	Novel chemotherapy (INT230-6)	180
NCT03849469	I	Recruiting	Bispecific antibody against CTLA-4 and LAG-3, combination bispecific antibody and immune checkpoint inhibitor (PD-1)	N/A	242
NCT04641871	I	Active, not recruiting	Combination immune checkpoint inhibitors (PD-1 with LAG-3 or TIM-3)	N/A	200
NCT04672434	I	Recruiting	Combination immune checkpoint inhibitors (PD-1 with CD73)	N/A	100
NCT03872947	I	Recruiting	Monoclonal antibody against undisclosed tumor-associated antigen with immune checkpoint inhibitors (PD-1 or CTLA-4)	Imiquimod Cream, Irinotecan, Leucovorin, 5-FU, Gemcitabine, Cisplatin, Carboplatin, Ramucirumab, Paclitaxel	75
NCT03801083	II	Recruiting	TIL adoptive cell transfer	N/A	59
NCT03633773	I/II	Recruiting	MUC-1 CAR-T cell	N/A	9
NCT04951141	I	Recruiting	GPC3 CAR-T cells	N/A	10
NCT01868490	I/II	Recruiting	CIK adoptive cell transfer	N/A	13
NCT03942328	I	Recruiting	Autologous dendritic cells	External Beam Radiation Therapy, Pneumococcal 13-valent Conjugate Vaccine	26
NCT04853017	I/II	Recruiting	Peptide vaccine against KRAS mutations	N/A	159

**Table 3:** Clinical trials currently evaluating immunotherapies in GBC. List of clinical trials involving combination immune checkpoint inhibitors, adoptive cell transfer, and other innovative immunotherapies in GBC. Source: clinicaltrials.gov (28 July 2021). GBC, gallbladder carcinoma; TIL, tumor-infiltrating lymphocyte; CRISPR, clustered regularly interspaced short palindromic repeats.

Trial Identifier	Phase	Status	Immunotherapy	Co-Treatments	Participants
NCT02834013	II	Recruiting	Combination immune checkpoint inhibitors (CTLA-4 with PD-1)	N/A	818
NCT03473574	II	Active, not recruiting	Combination immune checkpoint inhibitors (CTLA-4 with PD-1)	Gemcitabine, Cisplatin	128
NCT02821754	II	Recruiting	Combination immune checkpoint inhibitors (CTLA-4 with PD-1)	Transarterial catheter chemoembolization, radiofrequency ablation, cryoablation	90
NCT04641871	I	Active, not recruiting	Combination immune checkpoint inhibitors (PD-1 with LAG-3 or TIM-3)	N/A	200
NCT04672434	I	Recruiting	Combination immune checkpoint inhibitors (PD-1 with CD73)	N/A	100
NCT03801083	II	Recruiting	TIL adoptive cell transfer	N/A	59
NCT04426669	I/II	Recruiting	CRISPR gene editing in TIL adoptive cell transfer	Aldesleukin, Cyclophosphamide, Fludarabine	20
NCT04853017	I/II	Recruiting	Peptide vaccine against KRAS mutations	N/A	159

## Data Availability

Data are available on request.
